# A novel phospho-modulatory mechanism contributes to the calcium-dependent regulation of T-type Ca^2+^ channels

**DOI:** 10.1038/s41598-019-52194-6

**Published:** 2019-10-30

**Authors:** Jean Chemin, Tamara Timic Stamenic, Magalie Cazade, Jodie Llinares, Iulia Blesneac, Slobodan M. Todorovic, Philippe Lory

**Affiliations:** 1 IGF, CNRS, INSERM, University of Montpellier, Montpellier, France; 2 LabEx’Ion Channel Science and Therapeutics’, 34094 Montpellier, France; 3 Department of Anesthesiology, University of Colorado, Aurora, CO 80045 USA

**Keywords:** Cellular neuroscience, Ion channels in the nervous system

## Abstract

Ca_v_3 / T-type Ca^2+^ channels are dynamically regulated by intracellular Ca^2+^ ions, which inhibit Ca_v_3 availability. Here, we demonstrate that this inhibition becomes irreversible in the presence of non-hydrolysable ATP analogs, resulting in a strong hyperpolarizing shift in the steady-state inactivation of the residual Ca_v_3 current. Importantly, the effect of these ATP analogs was prevented in the presence of intracellular BAPTA. Additional findings obtained using intracellular dialysis of inorganic phosphate and alkaline phosphatase or NaN_3_ treatment further support the involvement of a phosphorylation mechanism. Contrasting with Ca_v_1 and Ca_v_2 Ca^2+^ channels, the Ca^2+^-dependent modulation of Ca_v_3 channels appears to be independent of calmodulin, calcineurin and endocytic pathways. Similar findings were obtained for the native T-type Ca^2+^ current recorded in rat thalamic neurons of the central medial nucleus. Overall, our data reveal a new Ca^2+^ sensitive phosphorylation-dependent mechanism regulating Ca_v_3 channels, with potentially important physiological implications for the multiple cell functions controlled by T-type Ca^2+^ channels.

## Introduction

Voltage-gated Ca^2+^ channels (VGCCs) are widely expressed in neurons, heart and muscles where they contribute to many critical physiological functions including cellular excitability but also muscle contraction, neurotransmitter release and gene expression^1^. An alteration in VGCC function leads to several diseases, including epilepsy, ataxia, chronic pain as well as cardiac pathologies^1,2^. Therefore, VGCC activity is tightly regulated by multiple intracellular pathways, including Ca^2+^ ions, which provide an important feedback control of Ca^2+^ homeostasis^3,4^.

VGCCs are divided into three families: the L-type channels (Ca_v_1 family); the neuronal N-, P/Q-, and R-type channels (Ca_v_2 family); and the T-type channels (Ca_v_3 family)^5^. The Ca_v_3 channels display unique electrophysiological features including a low-voltage-activated Ca^2+^ current, fast inactivation kinetics and a strong steady-state inactivation at physiological resting potentials^6,7^. These electrophysiological properties of T-type channels allow the generation of low-threshold spikes in neurons subsequent to transient membrane hyperpolarization^6,7^. Low-threshold spikes mediate rebound burst firing, which have important physiological implication, especially in thalamo-cortical circuit where T-type channels control transition between awake and sleep states^6–9^. T-type channels are also implicated in several neuronal disorders including epilepsy, chronic pain, autism, schizophrenia and cerebellar ataxia^2,8,10–12^. Consequently, the understanding of T-type channel regulation is of great physiological importance.

In contrast with Ca_v_1 and Ca_v_2 VGCCs, for which the regulation by intracellular Ca^2+^ has been extensively studied^3,4,13,14^, the regulation of Ca_v_3 channels by Ca^2+^ ions has been underappreciated until recently^15–18^. We have previously shown that the Ca_v_3.3 current (and to a lesser extend the Ca_v_3.1 current but not the Ca_v_3.2 current) is inhibited at a fast frequency of stimulation, providing an important feedback control^15^. This inhibition is associated with faster current inactivation kinetics and a negative shift in the steady-state inactivation of the Ca_v_3.3 current. Interestingly, these effects are observed when recording Ca_v_3.3 inward current but not Ca_v_3.3 outward current indicating that a role of a voltage-dependent inactivation is unlikely^15^. Furthermore the current inhibition is abolished in the presence of intracellular BAPTA but not EGTA, indicating the involvement of submembrane Ca^2+^ ions. However, Ca^2+^ ions have no effect on the Ca_v_3.3 current recorded in cell-free inside-out patches indicating the necessity of an intracellular transduction pathway that takes place in the intact cells. For the Ca_v_1 and Ca_v_2 VGCCs, several studies have indicated that, depending of the amount of the Ca^2+^ entry, the Ca^2+^-dependent regulation of these channels mainly involves activation of calmodulin^19–22^ and calcineurin^23,24^ as well as Ca^2+^-dependent endocytosis of the channels^21,25,26^. We therefore investigated whether any of these Ca^2+^-dependent processes could be involved in the high frequency stimulation-induced inhibition of Ca_v_3.3 current or its recovery following termination of the stimulation. Our results demonstrate that this Ca_v_3.3 current modulation does not depend on calmodulin, calcineurin or endocytosis of the channels but is regulated by a phosphorylation-dependent process. We found that a Ca^2+^-dependent phosphorylation controls the availability of Ca_v_3 channels by adjusting the voltage-dependence of the steady-state inactivation curve. Similar findings were observed on native T-type channels expressed in the central medial nucleus of the thalamus, in which T-type channels mediate rebound burst firing^27^. Hence, our findings strongly suggest the involvement of a novel Ca^2+^ / phosphorylation-dependent transduction pathway that finely adjusts the T-type channel properties necessary for proper physiological function.

## Results

**Figure 1:**
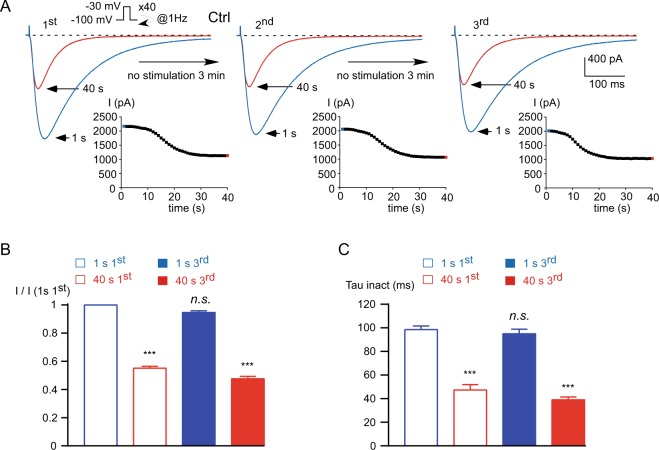
The Ca_v_3.3 current inhibition induced by stimulation at 1 Hz frequency (activity-dependent) is reversible and reproducible. (**A**) Typical examples of Ca_v_3.3 currents elicited by a test pulse stimulation at −30 mV (450 ms duration) applied at a frequency of 1 Hz from a holding potential (HP) of −100 mV. The traces obtained at the beginning of the stimulation (1 s) and after 40 s stimulation are indicated in blue and red, respectively. Stopping the stimulation for 3 minutes allows the recovery of the Ca_v_3.3 current. The protocol was applied 3 times and the time course of the current amplitude is indicated as an inset. (**B**) Amplitude of the Ca_v_3.3 current at the beginning (1 s) and after 40 s obtained at the 1^st^ and the 3^rd^ stimulation normalized to the initial current amplitude obtained at the beginning of the 1^st^ stimulation: I (1 s 1^st^), n = 21. (**C**) Inactivation kinetics of the Ca_v_3.3 current at the beginning of the stimulation (1 s) and after 40 s obtained for the 1^st^ and the 3^rd^ stimulation (n = 21). Asterisks indicate significant difference as compared to the initial current (1 s 1^st^) amplitude (**B**) and inactivation kinetics (**C**). (*n*.*s*.: non-significant).

At high frequency stimulation, the Ca_v_3.3 current displays a Ca^2+^-dependent inhibition that is dynamically regulated^15^. During stimulations applied at 1 Hz frequency, the Ca_v_3.3 current decreased ~45% from its initial value after 40 seconds (*p* < 0.001, Fig. 1A,B) and this decrease was associated with faster inactivation kinetics (*p* < 0.001, Fig. 1C). Stopping the stimulation for 3 minutes allowed a full recovery of Ca_v_3.3 current amplitude, after which Ca_v_3.3 current decrease at fast stimulation could be reproduced several times (Fig. 1A, 2^nd^ and 3^rd^). Applying this protocol 3 times (Fig. 1A) allowed us to quantify both the current inhibition and its recovery after the 1^st^ and the 3^rd^ stimulation. Quantification of current inhibition and current recovery was calculated as the ratio of the current amplitude obtained at the 3^rd^ stimulation before and after the 1 Hz stimulation to the initial current amplitude obtained at the 1^st^ stimulation (I 1 s 1^st^; Fig. 1B). Taking advantage of the reversibility and reproducibility of the Ca^2+^-dependent inhibition of the Ca_v_3.3 current, we investigated the mechanisms underlying current decrease as well as current recovery.

It has been demonstrated that a prolonged Ca^2+^ entry induces an internalization of the L-type channels via endocytosis, possibly leading to degradation in the lysosome^21,25,26^. We tested whether this pathway was implicated in the inhibition of Ca_v_3.3 current during fast stimulation (Supplementary Fig. 1). Endocytosis was disrupted by expressing a dominant negative mutant of dynamin 1 (Dyn 1 K44A), a GTPase required for the formation of endocytic vesicles from the plasma membrane and implicated in the endocytosis of the L-type channel^25,28^. Expression of the Dyn 1 K44A mutant (Supplementary Fig. 1B) did not alter the inhibition of the Ca_v_3.3 current during fast stimulation (~45%, *p* < 0.001, red bars in Supplementary Fig. 1E) as compared to WT dynamin or the control condition (*p* > 0.05, Supplementary Fig. 1A,E). In addition, the recovery of the Ca_v_3.3 current was not different in cells expressing the Dyn 1 K44A mutant (blue bars in Supplementary Fig. 1E) as compared to WT dynamin or the control condition (*p* > 0.05, Supplementary Fig. 1A,E). Similar findings were obtained after expression of the adaptor protein 2 (AP-2), a central player in clathrin-mediated endocytosis^29^, which did not modify current inhibition (~45%, *p* < 0.01) or its recovery (Supplementary Fig. 1C,E), as compared to the control condition (*p* > 0.05, Supplementary Fig. 1E). In addition, we have treated the cells with the lysosomal inhibitor bafilomycin (100 nM, 3–5 hours), using the same protocol as described for L-type channels^26^. We found that bafilomycin did not affect Ca_v_3.3 current inhibition at a high frequency of stimulation, nor its recovery (Supplementary Fig. 1D,E). The analysis of the Ca_v_3.3 current also indicated no significant effect of dynamin K44A, AP2 or bafilomycin treatment on current inactivation kinetics at the beginning as well as at the end of the fast stimulation protocol, as compared to the respective control condition (*p* > 0.05, n = 5–6).

**Figure 2:**
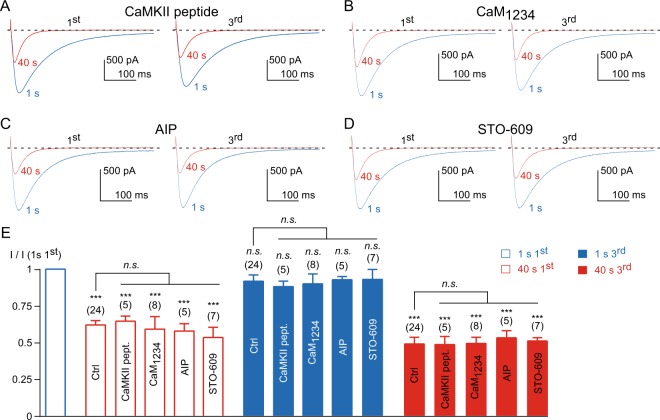
Calmodulin and CaMKII are not involved in the activity-dependent Ca_v_3.3 current inhibition. (**A**–**D**) Examples of Ca_v_3.3 currents elicited by the 1 Hz stimulation protocol during 40 s for the 1^st^ and the 3^rd^ stimulation in cells dialyzed with 100 µM CaMKII peptide (**A**), in cells expressing CaM_1234_ (**B**), in cells dialyzed with 100 µM autocamtide-2-related inhibitory peptide, AIP (**C**), and in cells treated for 2 hours with 1 µM STO-609 (**D**). (**E**) Amplitude of the Ca_v_3.3 current at the beginning (1 s) and after 40 s obtained during the 1^st^ and the 3^rd^ stimulation normalized to the initial current amplitude obtained at the beginning of the 1^st^ stimulation I (1 s 1^st^). Asterisks indicate significant difference in the current amplitude, as compared to the initial current amplitude. In addition, the current inhibition (red bars) and recovery (blue bars), as function of the treatment, was statistically compared to the respective control condition, as indicated. The number of cells tested is indicated into brackets. (*n*.*s*.: non-significant).

Calmodulin (CaM) is important for the modulation of the L-type current^19–22^ and has emerged recently as a modulator of T-type channels, including Ca_v_3.3^16–18^. To investigate this pathway we first used a CaMKII peptide (100 µM CaMKII 290–309) that binds CaM and inhibits its function and additional calmodulin-requiring enzymes. Dialysis of this peptide did not modify the Ca_v_3.3 current inhibition at 1 Hz frequency of stimulation, nor its recovery (*p* > 0.05, as compared to the control condition, Fig. 2A,E). We next used a CaM mutant, CaM_1234_, which is unable to bind Ca^2+^ and was previously used to demonstrate the CaM regulation of the L-type channel^19,20^. Expression of this mutant was also without significant effect on Ca_v_3.3 modulation (Fig. 2B,E), indicating that CaM was not involved in the decrease of Ca_v_3.3 current induced by stimulation at a high frequency or in its recovery. Similar results were obtained using the CaMKII inhibitory peptide (autocamtide-2-related inhibitory peptide, AIP, 100 µM intrapipette) and STO-609, a CaMKK inhibitor (1 µM incubation for 2 hours; Fig. 2C–E). In addition, the current inactivation kinetics at the beginning and at the end of the fast stimulation protocol were not significantly modified by CaMKII and AIP peptides or CaM_1234_ and STO-609, as compared to the respective control condition (*p* > 0.05, n = 5–8). However, we found that CaM_1234_ expression induced a negative shift ~5.5 mV in the steady-state inactivation of the Ca_v_3.3 current (n = 8, *p* < 0.005) compared to the control condition (n = 5), as recently reported for the Ca_v_3.2 current^17^.

**Figure 3:**
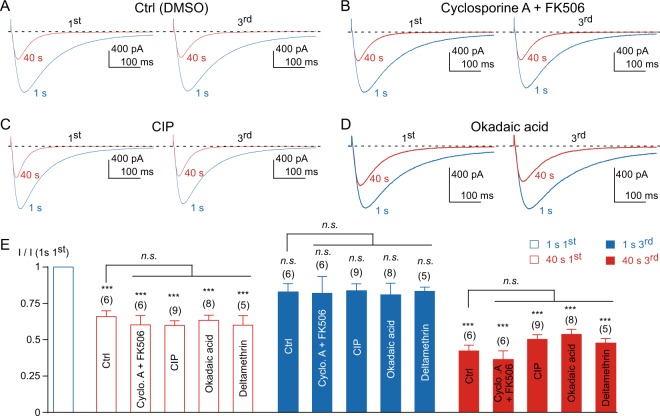
Calcineurin and okadaic acid sensitive phosphatases are not involved in the activity-dependent Ca_v_3.3 current inhibition. (**A–D**) Examples of Ca_v_3.3 currents elicited by the 1 Hz stimulation protocol during 40 s for the 1^st^ and the 3^rd^ stimulation in the control condition (**A**), in the presence of 10 µM cyclosporin A and 10 µM FK506 (**B**), in the presence of 100 µM calcineurin inhibitory peptide (CIP) (**C**), and in the presence of 1 µM okadaic acid (**D**). (**E**) Amplitude of the Ca_v_3.3 current at the beginning (1 s) and after 40 s obtained at the 1^st^ and the 3^rd^ stimulation normalized to the initial current amplitude I (1 s 1^st^). Asterisks indicate significant difference in the current amplitude, as compared to the initial current amplitude. In addition, the current inhibition (red bars) and recovery (blue bars), as function of the treatment, was statistically compared to the respective control condition, as indicated. The number of cells tested is indicated into brackets. (*n*.*s*.: non-significant).

It was also demonstrated that during 1 Hz frequency stimulation, the L-type current decreases similarly to the Ca_v_3.3 current and that this effect is mediated in part by calcineurin^23,24^. We have therefore tested whether this pathway was implicated in the Ca_v_3.3 current decrease. Cells were treated with a combination of cyclosporin A and FK506 (10 µM each) before patch-clamp experiments, a treatment that prevents the decrease and the calcium-dependent inactivation (CDI) of the L-type current^23,24^. We found that 1–2 hours incubation with cyclosporin A and FK506 did not affect the decrease of the Ca_v_3.3 current, nor its recovery, as compared to vehicle (DMSO)-treated cells (*p* > 0.05, Fig. 3A,B,E). Similarly, inclusion of the calcineurin inhibitory peptide (CIP, 100 µM in the patch pipette), which also prevents L-type current decrease and CDI^23,24^, did not affect the Ca_v_3.3 current modulation (Fig. 3C,E). We also found that both inhibition and recovery of the Ca_v_3.3 current were unaffected by okadaic acid (100 nM overnight incubation and 1 µM in the patch pipette) or by deltamethrin (10 µM in the patch pipette) (Fig. 3D,E). In addition, none of the compounds tested modified the current inactivation kinetics at the beginning and at the end of the fast stimulation protocol (*p* > 0.05, as compared to the control condition, n = 5–9). Similarly, no significant change on the steady-state inactivation of the Ca_v_3.3 current was observed (*p* > 0.05, as compared to the control condition, n = 7–14). Overall, these findings strongly suggest that neither endocytosis nor CaM-dependent pathways nor calcineurin play a significant role in the Ca_v_3.3 current modulation at a high frequency of stimulation, which therefore requires different molecular mechanisms than those previously described for the L-type channel.

**Figure 4:**
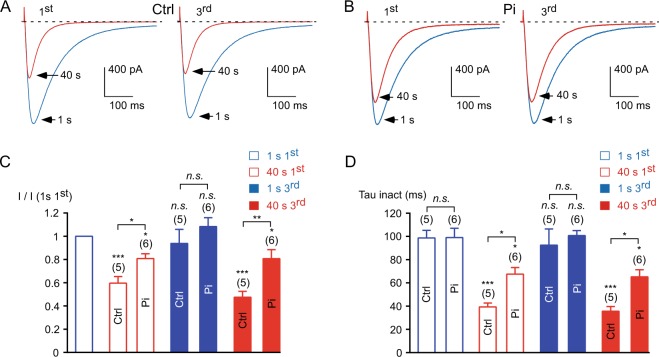
The activity-dependent Ca_v_3.3 current inhibition is reduced in the presence of inorganic phosphate (Pi). (**A**,**B**) Examples of Ca_v_3.3 currents elicited by the 1 Hz stimulation protocol during 40 s for the 1^st^ and the 3^rd^ stimulation in cells dialyzed with 15 mM KCl, Ctrl (**A**), and in cells dialyzed with 10 mM KH_2_PO_4_, Pi (**B**). (**C**) Amplitude of the Ca_v_3.3 current at the beginning (1 s) and after 40 s obtained at the 1^st^ and the 3^rd^ stimulation normalized to the initial current amplitude I (1 s 1^st^). (**D**) Inactivation kinetics of the Ca_v_3.3 current at the beginning of the stimulation (1 s) and after 40 s stimulation obtained for the 1^st^ and the 3^rd^ stimulation. Asterisks indicate significant difference in the current amplitude (**C**) and inactivation kinetics (**D**), as compared to the initial current. In addition, variation in the current amplitude (**C**) and in inactivation kinetics (**D**) as function of the Pi treatment was statistically compared to the respective control condition, as indicated. The number of cells tested is indicated into brackets. (*n*.*s*.: non-significant).

In order to investigate further the role of a phosphorylation mechanism in Ca_v_3.3 current modulation, we used inorganic phosphate to compete with phosphatase activities^30,31^. Inorganic phosphate (Pi, 10 mM KH_2_PO_4_) was applied via the patch pipette 10 minutes before stimulation of the Ca_v_3.3 current. In the presence of intracellular Pi, fast stimulation induced a slight current decrease ~20% (*p* < 0.05, Fig. 4B,C) whereas the current decrease was ~45% in the control condition (15 mM KCl in the patch pipette, *p* < 0.001, Fig. 4A,B). In average, the current decrease in the presence of Pi was ~35% less than the control value at the 3^rd^ stimulation (*p* < 0.01; Fig. 4C), whereas there was a slight but not significant increase in current recovery (Fig. 4C). In addition, during the stimulation at 1 Hz frequency, the acceleration in the inactivation kinetics was less pronounced in the presence of intracellular Pi as compared to the control condition (*p* < 0.05, Fig. 4D). It should be noted that in addition to competing with phosphatase activities, KH_2_PO_4_ may bind Ca^2+^ and therefore may act as a Ca^2+^ buffer. However, we observed a similar decrease in Ca_v_3.3 current in the cells dialyzed with an intracellular solution containing 20 mM EGTA, as compared to our standard recording condition using 10 mM EGTA (*p* > 0.5, n = 17), suggesting that the Pi effect does not rely on its Ca^2+^ buffer property. Moreover, these results suggest that a Ca^2+^-driven phosphatase might be responsible for the current decrease (see schematic representation in Supplementary Fig. 7). In agreement with the involvement of a phosphorylation mechanism, inclusion of recombinant alkaline phosphatase (AP, 100 U/ml) in the patch pipette^32^ “mimicked” the inhibition of the Ca_v_3.3 current at 1 Hz frequency stimulation (Supplementary Fig. 2A,B) and induced an acceleration in the current inactivation kinetics (Supplementary Fig. 2A–C). Importantly, the remaining current after AP treatment was stable during the 1 Hz frequency stimulation protocol (Supplementary Fig. 2A,B). In addition, the steady-state inactivation of the Ca_v_3.3 current was largely shifted towards negative potentials after AP dialysis (~25 mV, *p < *0.001; Supplementary Fig. 2D).

**Figure 5:**
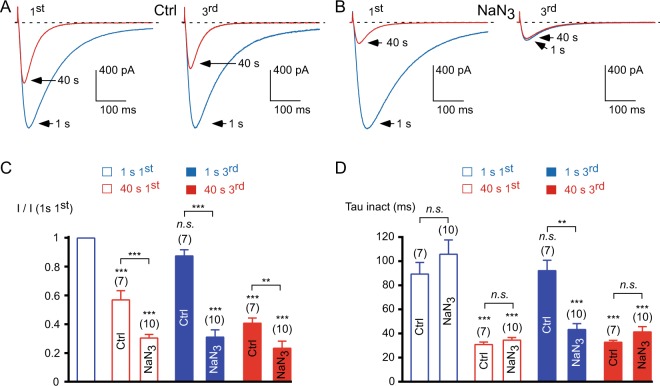
The recovery of the Ca_v_3.3 current is abolished in the presence of sodium azide. (**A**,**B**) Examples of Ca_v_3.3 currents elicited by the 1 Hz stimulation protocol during 40 s for the 1^st^ and the 3^rd^ stimulation in the control condition, Ctrl (**A**), and in cells treated with sodium azide, NaN_3_ 10 mM for 2 hours (**B**). (**C**) Amplitude of the Ca_v_3.3 current at the beginning (1 s) and after 40 s obtained at the 1^st^ and the 3^rd^ stimulation normalized to the initial current amplitude I (1 s 1^st^). (**D**) Inactivation kinetics of the Ca_v_3.3 current at the beginning of the stimulation (1 s) and after 40 s stimulation obtained for the 1^st^ and the 3^rd^ stimulation. Asterisks indicate significant difference in the current amplitude (**C**) and inactivation kinetics (**D**), as compared to the initial current. In addition, variation in the current amplitude (**C**) and in inactivation kinetics (**D**) as function of the NaN_3_ treatment was statistically compared to the respective control condition, as indicated. The number of cells tested is indicated into brackets. (*n*.*s*.: non-significant).

**Figure 6:**
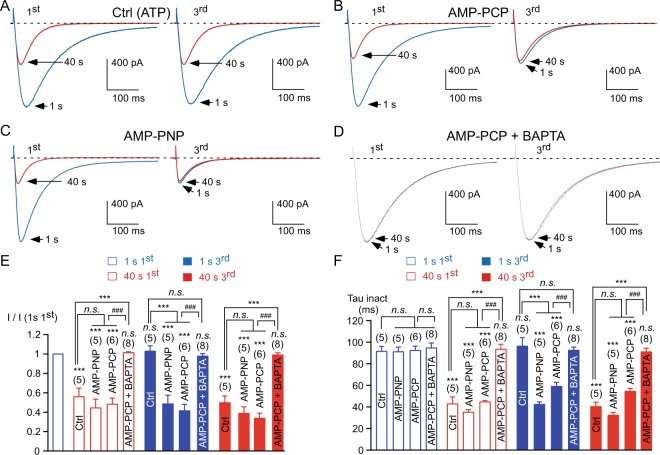
Intracellular ATP is required for the recovery of the Ca_v_3.3 current. (**A–D**) Examples of Ca_v_3.3 currents elicited by the 1 Hz stimulation protocol during 40 s for the 1^st^ and the 3^rd^ stimulation in cells dialyzed with 3 mM ATP, Ctrl (**A**), in cells dialyzed with 3 mM AMP-PCP (**B**), in cells dialyzed with 3 mM AMP-PNP (**C**) and in cells dialyzed with 3 mM AMP-PCP and 30 mM BAPTA (**D**). (**E**) Amplitude of the Ca_v_3.3 current at the beginning (1 s) and after 40 s obtained at the 1^st^ and the 3^rd^ stimulation normalized to the initial current amplitude I (1 s 1^st^). (**F**) Inactivation kinetics of the Ca_v_3.3 current at the beginning of the stimulation (1 s) and after 40 s stimulation obtained for the 1^st^ and the 3^rd^ stimulation. Asterisks indicate significant difference in the current amplitude (**E**) and inactivation kinetics (**F**), as compared to the initial current. In addition, variation in the current amplitude (**E**) and in inactivation kinetics (**F**) as function of the treatment was statistically compared to the respective control condition, as indicated. The hash signs indicate statistical difference between AMP-PCP and AMP-PCP + BAPTA. The number of cells tested is indicated into brackets. (*n*.*s*.: non-significant).

Considering that current inhibition could result from a phosphatase activity, we investigated whether a putative kinase activity might be implicated in current recovery (see schematic representation in Supplementary Fig. 7). To this purpose, the cells were incubated in the presence of 10 mM sodium azide (NaN_3_), which inhibits mitochondrial function^33^ and thereby results in a depletion of intracellular ATP (Fig. 5A,B). In the presence of NaN_3_ (and in the absence of ATP in the patch pipette), the 1 Hz stimulation induced a strong inhibition of the Ca_v_3.3 current (~70%, *p* < 0.001, Fig. 5C), which is significantly larger than that observed in the control condition (*p* < 0.001, Fig. 5C). Interestingly the recovery of the current at the 3^rd^ stimulation was completely abolished after NaN_3_ treatment and the residual current displayed fast inactivation kinetics (*p* < 0.001, Fig. 5C,D), suggesting that the recovery of the Ca_v_3.3 current relies on an ATP-dependent mechanism. We therefore investigated whether the hydrolysis of the phosphate group from ATP is needed for current recovery. To this end we tested the effect of non-hydrolysable ATP analogs (Fig. 6). When intracellular ATP in the patch pipette was replaced by AMP-PCP, the inhibition of the Ca_v_3.3 current (~55%, *p* < 0.001) at 1 Hz frequency stimulation was not significantly modified as compared to the control condition (*p* > 0.05, Fig. 6A,B,E), but the recovery of the Ca_v_3.3 current was completely abolished (*p* < 0.001; Fig. 6A,B,E). Similar findings were observed using another non-hydrolysable ATP analog, AMP-PNP, which completely abolished the recovery of the Ca_v_3.3 current (*p* < 0.001; Fig. 6C,E) but did not modify current inhibition as compared to the control condition (*p* > 0.05, Fig. 6E). In the presence of AMP-PCP or AMP-PNP, the remaining current after stimulation at high frequency displayed fast inactivation kinetics (*p* < 0.001, Fig. 6F). Because the depression of the current recovery may represent a spontaneous loss of channel activity (rundown) in the absence of intracellular ATP, we investigated the effect of AMP-PCP on the maintenance of Ca_v_3.3 current. To this purpose, Ca_v_3.3 currents were recorded during 30 minutes at a low frequency of stimulation (one stimulation each 180 s / 0.0055 Hz) in the presence of intracellular ATP or intracellular AMP-PCP (Supplementary Fig. 3). At this low frequency of stimulation, there was no significant difference in Ca_v_3.3 current rundown between control and AMP-PCP dialyzed cells (Supplementary Fig. 3A). On average channel activity was 94 ± 6% (n = 6) and 89 ± 6% (n = 6) of the initial value after 30 minutes of dialysis with 3 mM intracellular ATP and with 3 mM intracellular AMP-PCP, respectively. Thus, the decrease in Ca_v_3.3 current recovery was not due to faster rundown of the current but to ATP dependency of current recovery from 1 Hz frequency stimulation. Accordingly, after 30 minutes of dialysis, the application of the 1 Hz stimulation protocol induced a decrease in the Ca_v_3.3 current, which was irreversible in AMP-PCP dialyzed cells (*p* < 0.001, Supplementary Fig. 3A). Importantly, in the presence of intracellular BAPTA (instead EGTA), which prevents a localized increase in submembrane Ca^2+^ and the Ca^2+^-dependent inhibition of the Ca_v_3.3 current^15^, we found that AMP-PCP did not produce current inhibition (*p* > 0.05, Fig. 6D–F) strongly indicating that the effect of non-hydrolysable ATP analogs relies on a Ca^2+^-dependent phosphorylation mechanism. It should be noted however, that NaN_3_ and non-hydrolysable ATP analogs may also interfere with mitochondrial function and could lead to an irreversible oxidative stress that may underlie the Ca_v_3.3 current inhibition observed at fast frequency of stimulation. In order to investigate the potential role of the redox status in the current inhibition, the cells were dialyzed with an intracellular medium containing 1 mM dithiothreitol (DTT). After dialysis of DTT, the 1 Hz stimulation protocol induced inhibition of Ca_v_3.3 current amplitude (~40%, *p* < 0.001, Supplementary Fig. 4B,C) that was associated with an acceleration of current inactivation kinetics (*p* < 0.001, Supplementary Fig. 4D). These effects were not different to those observed in the control condition (*p* > 0.05, Supplementary Fig. 4C,D). Similarly, current recovery was unaffected (Supplementary Fig. 4C), suggesting that current modulation does not depend on the redox status of the Ca_v_3.3 channel. We noted however that in the presence of DTT, the inactivation kinetics were slower at the beginning of the 1^st^ stimulation as compared to the control condition (*p* < 0.05, n = 7) but this effect was not significant at the 3^rd^ stimulation (Supplementary Fig. 4D).

Several pathways regulating Ca_v_3 current were identified, including PKA^34–36^, PKC^35,37,38^, Rho-associated kinase^39^, phospholipase C (PLC^40^), and Gβγ^36,41^. We have tested whether these pathways could be implicated in the Ca_v_3.3 modulation at high frequency of stimulation using dibutyryl-cAMP (db-cAMP, a PKA activator), phorbol myristate acetate (PMA, a PKC activator), chelerythrine and bisindolylmaleimide IX (BIM IX, both PKC inhibitors), fasudil (a Rho-associated kinase inhibitor), U73122 and edelfosine (both PLC inhibitors) and pertussis toxin (PTX) or expression of the βARK-Ct (both inhibiting Gβγ). In the presence of these compounds, the inhibition (~50%, *p* < 0.01, Supplementary Fig. 5A) of the Ca_v_3.3 current was not different to that observed in the control condition (*p* > 0.05, Supplementary Fig. 5A). Importantly, these inhibitors did not modify the recovery of the Ca_v_3.3 current as compared to the control condition (*p* > 0.05, Supplementary Fig. 5B). Similarly, the inhibition of PI3K and PI4K with wortmannin or the inhibition of SERCA^42^ with cyclopiazonic acid (CPA) was without effect on both inhibition and recovery of the Ca_v_3.3 current (*p* > 0.05 as compared to the control condition, Supplementary Fig. 5A,B). Collectively, these results indicate that the Ca_v_3.3 current modulation at high frequency of stimulation involves a yet undescribed transduction pathway. We next tested several other kinases that could possibly modulate Ca_v_3.3 current. We found that the presumed inhibition of PDK1 with OSU-03012, of AKT with AKT1/2 inhibitor, of PLK1/3 with GW843682X and BI 2536, of RSK with BI-D1870, of TAK1 with 5Z-oxozeanol, of MNK1 with CGP57380, of PYK2/FAK with PF-431396, of SGK with GSK 650394, of GSK3 with CT 99021, of SRC with SU6656, of eEF2 kinase with A484954 and of MLCK with ML7, was without significant effects on both inhibition and recovery of the Ca_v_3.3 current after the 1 Hz stimulation (*p* > 0.05 as compared to the values obtained in the control condition, Supplementary Fig. 6A,B).

**Figure 7:**
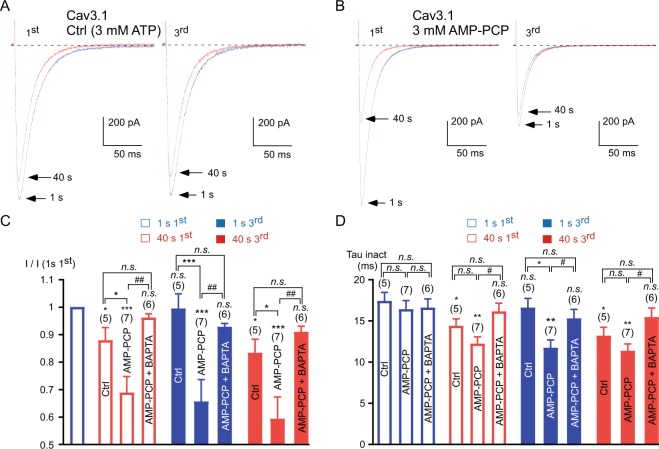
The recovery of the Ca_v_3.1 current after its inhibition by the 1 Hz stimulation required intracellular ATP. (**A**,**B**) Typical examples of Ca_v_3.1 currents elicited by the 1 Hz stimulation protocol during 40 s for the 1^st^ and the 3^rd^ stimulation in cells dialyzed with 3 mM ATP, Ctrl (**A**) and in cells dialyzed with 3 mM AMP-PCP (**B**). The Ca_v_3.1 currents were elicited using a 1 Hz test pulse stimulation at −30 mV (180 ms duration) from a HP of −100 mV. (**C**) Amplitude of the Ca_v_3.1 current at the beginning (1 s) and after 40 s obtained at the 1^st^ and the 3^rd^ stimulation normalized to the initial current amplitude I (1 s 1^st^). (**D**) Inactivation kinetics of the Ca_v_3.1 current at the beginning of the stimulation (1 s) and after 40 s stimulation obtained for the 1^st^ and the 3^rd^ stimulation. Asterisks indicate significant difference in the current amplitude (**C**) and inactivation kinetics (**D**), as compared to the initial current. Variation in the current amplitude (**C**) and in inactivation kinetics (**D**) as function of the treatment was statistically compared to the respective control condition, as indicated. The hash signs indicate statistical difference between AMP-PCP and AMP-PCP + BAPTA. The number of cells tested is indicated into brackets. (*n*.*s*.: non-significant).

We also investigated the ATP-dependency of the recombinant Ca_v_3.1 current during fast stimulations (Fig. 7). In the presence of intracellular ATP, the Ca_v_3.1 current decreased ~15% from its initial value during the 1 Hz frequency stimulation (*p* < 0.05; Fig. 7A,C) with an acceleration of its inactivation kinetics (*p* < 0.05; Fig. 7A,D). Importantly, the Ca_v_3.1 current recovery was almost total when the stimulation ceased for 3 minutes, as illustrated at the beginning of the 3^rd^ stimulation (Fig. 7A,C). In contrast, in AMP-PCP dialyzed cells, the Ca_v_3.1 current inhibition became irreversible (*p* < 0.001 as compared to the control condition, Fig. 7B,C) and the remaining current displayed faster inactivation kinetics (*p* < 0.01, Fig. 7B,D). In addition, Ca_v_3.1 current inhibition in the presence of AMP-PCP (~35% inhibition, *p* < 0.001) was more potent than that observed in ATP dialyzed cells for the 1^st^ and the 3^rd^ stimulation (*p* < 0.05, Fig. 7B,C), indicating that current inhibition is a very dynamic process. Importantly, in cells dialyzed with AMP-PCP + BAPTA, the stimulation at 1 Hz did not produce current inhibition and the inactivation kinetics remained unchanged (*p* > 0.05, Fig. 7C,D).

**Figure 8:**
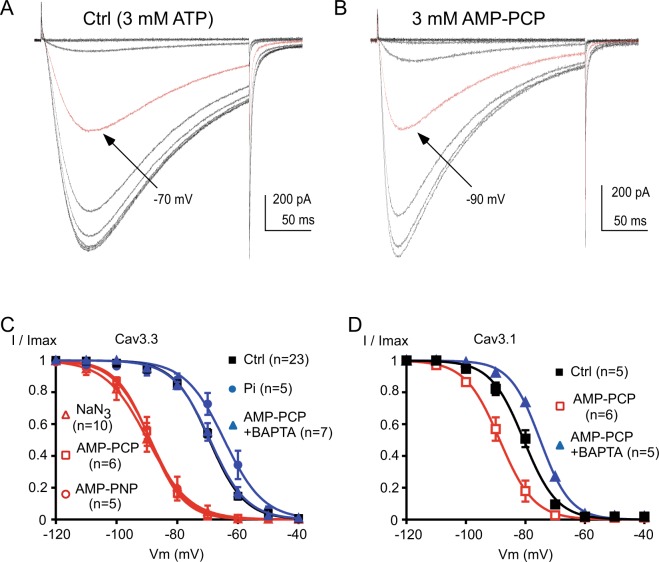
The steady-state inactivation of the Ca_v_3 current is dependent on intracellular ATP. (**A**,**B**) Typical Ca_v_3.3 currents recorded at −30 mV from holding potentials ranging from −120 to −40 mV (10 mV increment, 5 s duration) obtained in the control condition (**A**, 3 mM ATP) and in a cell dialyzed with 3 mM AMP-PCP (**B**). The red trace indicates the half-inactivation potential. (**C**) Steady-state inactivation curves of the Ca_v_3.3 current obtained from experiments presented in (**A**,**B**). (**D**) Steady-state inactivation curves of the Ca_v_3.1 current from cells dialyzed with ATP (control condition), AMP-PCP and AMP-PCP combined with BAPTA.

We have previously demonstrated that current inhibition relies mainly on a hyperpolarizing shift in the steady-state inactivation of the Ca_v_3.3 current^15^. Therefore, we have measured the steady-state inactivation of the Ca_v_3.3 current in the presence of non-hydrolysable ATP analogs. We found that, after the 3^rd^ 1 Hz stimulation in the presence of AMP-PCP, there was a strong hyperpolarizing shift ~20 mV in the steady-state inactivation of the Ca_v_3.3 current (Fig. 8A,B). In average, the V_0.5_ values of inactivation were −69.2 ± 0.6 mV in the control condition and −88.9 ± 1.1 mV in the presence of AMP-PCP (*p* < 0.001; Fig. 8C). Similar findings were obtained for AMP-PNP dialyzed cells and for NaN_3_ treated cells (Fig. 8C) since the V_0.5_ values of the steady-state inactivation curves were −88.7 ± 2.2 mV (*p* < 0.001) and −88.2 ± 2.7 mV (*p* < 0.001), respectively. In contrast, we observed only a slight depolarizing shift in the presence of Pi (V_0.5_ was −64.5 ± 2.3 mV; Fig. 8C), which was not significant. Importantly, we found that in the presence of intracellular BAPTA, AMP-PCP produced no significant shift in the steady-state inactivation curve of the Ca_v_3.3 current (*p* > 0.05, Fig. 8C). Similar findings were obtained for the recombinant Ca_v_3.1 current since the steady-state inactivation was significantly shifted towards negative potentials by ~8 mV in the presence of AMP-PCP and this shift was abolished in the presence of BAPTA (Fig. 8D). In average we found that the V_0.5_ values of the steady-state inactivation curves for the Ca_v_3.1 current were −80.6 ± 1.3 mV in the control condition, −88.3 ± 1.7 mV for the AMP-PCP dialyzed cells (*p* < 0.01) and −75.1 ± 0.4 mV for the cells dialyzed with AMP-PCP combined with BAPTA (*p* < 0.05 as compared to the control condition, and *p* < 0.0001 as compared to the AMP-PCP dialyzed cells).

**Figure 9:**
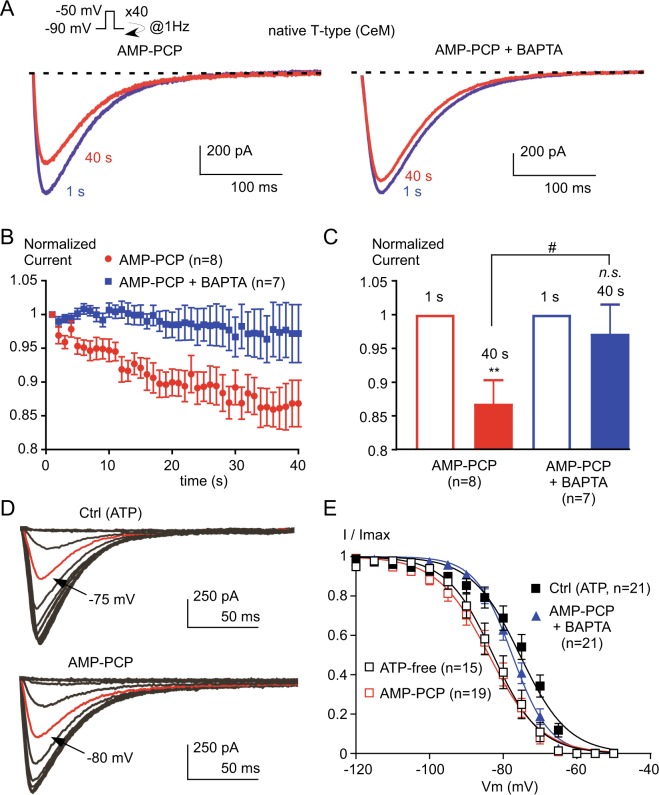
The native T-type current from neurons of rat central medial (CeM) nucleus is regulated by an interplay between a rise in intracellular calcium and a phosphorylation mechanism. (**A**) Typical T-type current elicited by stimulation at −30 mV applied at a frequency of 1 Hz from a holding potential of −90 mV obtained from CeM neurons dialyzed with either AMP-PCP or AMP-PCP combined with BAPTA. (**B**) Normalized current amplitude from 1 to 40 s. (**C**) Average normalized current amplitude recorded at 1 s and at 40 s. Asterisks indicate significant difference in the current amplitude at 40 s as compared to the current amplitude at 1 s. The hash signs indicate statistical difference between AMP-PCP and AMP-PCP + BAPTA. (**D**) Representative T-type current recorded at −50 mV from holding potentials ranging from −120 to −50 mV (5 mV increment, 3.6 s duration). CeM neurons were dialyzed with either ATP (control condition) or AMP-PCP. The red trace indicates the half-inactivation potential. (**E**) Average steady-state inactivation curves obtained from experiments presented in (**D**) for neurons dialyzed with ATP (control), AMP-PCP, AMP-PCP combined with BAPTA, and in absence of ATP (ATP-free).

We next explored whether a similar regulation of the native T-type current may occur in thalamic neurons. The effects of intracellular AMP-PCP and BAPTA were investigated in acute brain slices focusing on the T-type current expressed in the central medial nucleus (CeM) neurons of the thalamus, that are enriched in Ca_v_3.1 currents^27^. In the presence of AMP-PCP, the T-type current amplitude in CeM neurons decreased during stimulation at the frequency of 1 Hz (Fig. 9A,B) and reached ~15% inhibition after 40 s of stimulation (*p* < 0.01, Fig. 9B,C). This decrease in the T-type current amplitude was prevented by the substitution of intracellular EGTA with BAPTA suggesting the role of local increase in intracellular Ca^2+^ concentration in current inhibition (Fig. 9B,C). As observed for recombinant T-type channels, steady-state inactivation of the T-type current in CeM neurons was shifted towards the negative potentials in the presence of AMP-PCP (Fig. 9D,E). This shift in the steady-state inactivation was similar to the one observed when ATP was omitted in the intracellular solution (Fig. 9E), suggesting that the ATP effect relies mainly on a putative phosphorylation mechanism. In average, the V_0.5_ values of the steady-state inactivation curves were −75.4 ± 1.6 mV in the control condition with intracellular ATP, −82.3 ± 1.9 mV for the cells dialyzed with an ATP-free solution (*p* < 0.05) and −83.7 ± 1.65 mV for the cells dialyzed with AMP-PCP (*p* < 0.01, Fig. 9E). Importantly, the shift in the steady-state inactivation of the T-type current did not occur when the intracellular solution contained AMP-PCP in the presence of BAPTA (*p* > 0.05 as compared to the control conditions, Fig. 9E). Overall, these data strongly suggest that the availability of both recombinant and native T-type current depends on a phosphorylation process, which is regulated by the local concentration of intracellular calcium (see schematic representation in Supplementary Fig. 7).

## Discussion

Recently, we demonstrated that the availability of the Ca_v_3 channels can be dynamically tuned by changes in the intracellular Ca^2+^ concentration at the vicinity of these channels^15^. Here we describe that this Ca^2+^-induced modulation of the Ca_v_3 current likely relies on a phosphorylation mechanism. This modulation occurs for both Ca_v_3.3 and Ca_v_3.1 recombinant channels, as well as for native neuronal T-type channels. Importantly, this modulation of T-type channels by intracellular Ca^2+^ is novel among the Ca^2+^ channel family as it involves a phosphorylation process not related to calcineurin, endocytosis and calmodulin-dependent pathways.

The regulation of the high-voltage-activated Ca_v_1 and Ca_v_2 Ca^2+^ channels by a rise in intracellular Ca^2+^ has been extensively studied and serves as a reference^13,14,19–21,23–26^. For the L-type / Ca_v_1.2 channel a rise in intracellular Ca^2+^ induces complex effects depending on the amount of the calcium ions. Ca^2+^ entry via L-type channels first induces a Ca^2+^-dependent inactivation (CDI) characterized by an acceleration of the inactivation kinetics of the L-type current that is followed, if the stimulation persists, by a decrease/rundown of the current. It was established that both CDI^14,19–21,24^ and rundown^23,24^ of the L-type current are dependent on calmodulin activity. Clearly, the modulation of the Ca_v_3.3 current described here is independent of the calmodulin-dependent pathway, contrasting with the well-established Ca^2+^-dependent mechanisms regulating the L-type channels.

Importantly recent studies have revealed that calmodulin interacts with Ca_v_3 channels^16–18^. We have previously reported that the steady-state inactivation of the Ca_v_3.2 current was negatively shifted ~5 mV towards negative potentials in the presence of the CaM_1234_ mutant that does not bind Ca^2+^^17^, and we find here that this is also the case with the Ca_v_3.3 current. However, in the presence of this mutant, the inhibition of Ca_v_3.3 current at a high frequency of stimulation is preserved as well as the current recovery after the stopping of the stimulation. Similar findings were obtained using a CaMKII peptide that binds CaM and inhibits its function. In contrast with our results, another study found that in the presence of 1 µM intracellular Ca^2+^, the steady-state inactivation of the Ca_v_3.3 current is shifted ~6 mV towards negative potentials and this effect is occluded by the over-expression of the CaM_1234_ mutant^18^. This suggest that a prolonged and global increase in intracellular Ca^2+^^18^ may lead to the activation of distinct intracellular pathways, as compared to a transient and local increase in intracellular Ca^2+^, which occurs here during the fast stimulation of the Ca_v_3.3 current. The Ca^2+^/CaM-activated phosphatase calcineurin was also shown to interact with Ca_v_3.2 channels and Ca^2+^ entry via these channels induces the calcineurin-dependent activation of the transcription factor NFAT^43–45^. In addition, both L-type current decrease during fast stimulation and CDI were prevented after calcineurin inhibition^23,24^, suggesting that calcineurin may mediate inhibition of the Ca_v_3.3 current. However, prolonged treatment with several calcineurin inhibitors was without effect on Ca_v_3.3 current modulation, confirming a study on T-type currents in sensory neurons^46^. It was also demonstrated that Ca^2+^ entry via Ca_v_3 channels can promote a Ca^2+^-dependent CaM dissociation from the channels leading to CaMKII activation, which is blocked using the CaMKII inhibitory peptide AIP^16^. In addition, in the presence of micromolar concentrations of intracellular Ca^2+^, CaMKII can regulate Ca_v_3.2 current by phosphorylation of serine residues 1198 and 1153 in the channel’s 2–3 intracellular loop^47–49^. This regulation is abolished in the presence of the AIP peptide or by substitution of intracellular ATP by AMP-PNP^47^. However, this latter regulation of the Ca_v_3.2 current is clearly different from the one described here for the Ca_v_3.3 current, since Ca^2+^ / CaMKII activation promotes an increase (but not inhibition) of the Ca_v_3.2 current by promoting a negative shift in the activation curve without any effect on the steady-state inactivation. In addition, this Ca_v_3.2 regulation involves CaMKIIγc, which is not expressed in HEK-293 cells^47^. Accordingly, we found that the dialysis of the AIP peptide had no effect on both inhibition and recovery of the Ca_v_3.3 current. Therefore, although CaM/Calcineurin/CaMKII pathways interact with Ca_v_3 channels, our data strongly support the idea that the modulation of the Ca_v_3.3 current at a high frequency of stimulation requires additional Ca^2+^-dependent mechanisms.

Endocytosis could be another mechanism leading to the decrease of the Ca_v_3.3 current. Indeed, for the L-type channels, a prolonged current stimulation or activation of the ionotropic NMDA receptors induces the internalization of the channels and potentially their degradation in lysosomes or their recycling to the plasma membrane depending of the amount and the duration of the Ca^2+^ entry^21,25,26^. These studies have highlighted the crucial role of dynamin 1, a GTPase required for the formation of endocytic vesicles from the plasma membrane^28^, in the early steps of this process^21,25,26^. In the presence of a dominant negative mutant of dynamin 1 (Dyn 1 K44A), which abolishes the endocytosis of the L-type channel^25^, the inhibition of the Ca_v_3.3 current during 1 Hz stimulation is not affected as well as its recovery when the stimulation ceases. In addition, a role of lysosomal degradation is unlikely since Ca_v_3.3 current inhibition and its recovery are preserved after treatment of the cells with bafilomycin, which prevents degradation of the L-type channel^26^. Therefore, although the regulation by intracellular Ca^2+^ of L- and T-type channels share similar features, the mechanism underlying the Ca^2+^ dependent inhibition of T-type channels during fast stimulation is clearly different from the one described for L-type channels.

We found that the Ca_v_3.3 current modulation observed at a high frequency of stimulation likely relies on a phosphorylation mechanism (see schematic representation in Supplementary Fig. 7). Accordingly, the inhibition of the Ca_v_3.3 current during fast stimulation is decreased in a presence of intracellular Pi, which is a competitive inhibitor of phosphatase activities^30,31^. Conversely, sodium azide, a pharmacological agent that promotes the depletion of intracellular ATP by the inhibition of cytochrome oxidase^33^, the final enzyme in the mitochondrial electron transport chain, abolishes the recovery of the Ca_v_3.3 current after its inhibition induced by the fast stimulation. Therefore, we hypothesized that ATP is necessary in the recovery process. We used non-hydrolysable ATP analogs, AMP-PNP and AMP-PCP, which are competitive inhibitors of reactions requiring hydrolysable ATP^50^. The substitution of intracellular ATP with AMP-PNP or AMP-PCP, abolishes the recovery of the Ca_v_3.3 current, indicating the crucial role of a phosphorylation-dependent mechanism in the Ca^2+^-induced inhibition of the Ca_v_3.3 current. This depression of recovery in AMP-PCP dialyzed cells might be a consequence of a rundown of the Ca_v_3.3 current during the experiment leading to an apparent inhibition of recovery after fast stimulation. However, we found a similar rundown of the Ca_v_3.3 current when stimulated at a low frequency in ATP and in AMP-PCP dialyzed cells, indicating that the effect of AMP-PCP depends on the previous inhibition of the Ca_v_3.3 current by a fast stimulation. Accordingly, in the presence of intracellular BAPTA, which prevents a local submembrane rise in Ca^2+^ ^51–53^, AMP-PCP did not produce current inhibition indicating that the effect of non-hydrolysable ATP analogs relies on a Ca^2+^-dependent mechanism.

We used different pharmacological antagonists to examine the potential involvement of various intracellular pathways underlying the Ca_v_3.3 current modulation. Interestingly, the recovery of the Ca_v_3.3 current after fast stimulation does not depend of the previously identified kinases that modulated the Ca_v_3 current, including PKA, PKC and Rho-associated kinase^34–37,39^. The recovery of the Ca_v_3.3 current is also resistant to the inhibition of PI3K / PI4K, PDK1, AKT, PLK1/3, RSK, TAK1, PYK2/FAK, SGK, GSK3, SRC, eEF2 or MLCK. Therefore, further extensive biochemical and electrophysiological studies are needed to identify the precise signaling pathways that modulate the Ca_v_3.3 current.

We have previously demonstrated that a rise in submembrane Ca^2+^ ions induces a hyperpolarizing shift in the steady-state inactivation of the Ca_v_3.3 current, a regulation that is also found for the Ca_v_3.1 current^15^. Here we show that sodium azide, AMP-PNP and AMP-PCP treatment, induced a large ~20 mV hyperpolarizing shift in the steady-state inactivation of the Ca_v_3.3 current, confirming a common mechanism. Accordingly, AMP-PCP has no effect on the steady-state inactivation of the Ca_v_3.3 current in cells dialyzed with BAPTA. Regarding the Ca_v_3.1 current, its inhibition during stimulation at a high frequency in the presence of intracellular ATP is modest (~15%) compared to Ca_v_3.3, most likely due to its rapid inactivation kinetics leading to a moderate increase in intracellular Ca^2+^ ^54–56^. As observed for the Ca_v_3.3 current, the inhibition of the Ca_v_3.1 current is irreversible in the presence of intracellular AMP-PCP whereas the inhibition is prevented in the presence of BAPTA. In addition, the steady-state inactivation curve of the Ca_v_3.1 current is shifted by ~8 mV towards negative potentials in the presence of AMP-PCP. Therefore, our data reveal that the T-type channel modulation by intracellular Ca^2+^ has unusual features among the Ca^2+^ channel family both in its transduction and its underlying biophysical mechanisms.

Importantly, we found that the native Ca_v_3.1 T-type current recorded in neurons of the central medial nucleus (CeM) of the thalamus^27^, is subject to a similar Ca^2+^-dependent regulation. When stimulated at a frequency of 1 Hz, the T-type current in CeM neurons decreases ~15% in the presence of AMP-PCP and this effect is abolished in the presence of intracellular BAPTA. As observed for recombinant channels, this inhibition relies on a shift of the steady-state inactivation since the native T-type current displays a ~8 mV negative shift in the steady-state inactivation curve in the presence of AMP-PCP, which is abolished in the presence of BAPTA. This shift is similar to the one observed in cells dialyzed with an ATP-free solution, indicating that ATP effect relies on a phosphorylation mechanism. Interestingly, it was previously reported in thalamocortical neurons of the ventrobasal nucleus that the steady-state inactivation of the T-type current progressively shifts towards negative potentials during the dialysis of an ATP-free solution, indicating that the ATP-dependency of the T-type channel availability is not restricted to CeM neurons^57^. Overall, our study demonstrates that the availability of both recombinant and native T-type channels depends on a phosphorylation process regulated by the local intracellular Ca^2+^ concentration (Supplementary Fig. 7). This Ca^2+^-dependent inhibition of T-type channel would provide an important feedback mechanism during sustained neuronal activities to limit intracellular Ca^2+^ overload. It should be noted however that although our study highlights the role of a putative phosphorylation mechanism, whether the Ca_v_3 channel is directly phosphorylated or modulated via an intermediate protein remains an open question. Future molecular and biochemical studies will be necessary to clarify this important aspect and identify the key residues implicated in the Ca_v_3 current regulation.

By demonstrating that the availability of the T-type channels critically depends on an interplay between Ca^2+^ entry and the cellular phosphorylation status, we document a novel and important mechanism that tightly and dynamically controls T-type channel activity. Considering that T-type channels are widely expressed in the central nervous system where they contribute to the rebound burst firing activities^6,7^, and that an alteration in their activity is implicated in several neuronal disorders, including epilepsy, schizophrenia, autism, chronic pain and cerebellar ataxia^2,8,10–12^, this phosphorylation-dependent Ca^2+^-sensitive mechanism might be crucial to accurately balance the electrical and Ca^2+^ signaling of T-type channels both in their normal physiological responses and in disease states involving these channels.

## Methods

### Cell culture, transfection and electrophysiological recordings in tsA-201 cells

Cell culture and transfection of tsA-201 cells were performed as described previously^15^. Briefly, transfections were performed using jet-PEI (Ozyme, France) with a DNA mix (1.5 µg total) containing 0.5% of a GFP encoding plasmid and 99.5% of the pcDNA3.1 plasmid constructs that code for the human Ca_v_3.1 (isoform Ca_v_3.1a, Genbank accession number AF126966.1^58^) and human Ca_v_3.3 (isoform LT9, Genbank accession number AF393329.1^59^) channels. In some experiments, 1.5 µg of the plasmids that code for Dynamin 1, Dynamin 1 K44A, AP-2, CaM_1234_ or βARK-Ct was added to the DNA mix. Two days after transfection, tsA-201 cells were dissociated with Versene (Invitrogen, Fisher Scientific, France) and plated at low density for electrophysiological recordings, as described previously^15^. Macroscopic currents were recorded at room temperature using an internal solution containing (in mM): 140 CsCl, 10 EGTA, 10 HEPES, 3 Mg-ATP, 0.6 GTPNa, and 3 CaCl_2_ (pH adjusted to 7.25 with KOH, ~315 mOsm, ~100 nM free Ca^2+^ using the MaxChelator software, http://maxchelator.stanford.edu/) and an extracellular solution containing (in mM): 135 NaCl, 20 TEACl, 2 CaCl_2_, 1 MgCl_2_, and 10 HEPES (pH adjusted to 7.25 with KOH, ~330 mOsm).

### *In vitro* brain slice preparation from juvenile rats

Experimental procedures with animals were performed as described previously^27^ according to the protocol #B-111616 (02) 1E approved by the Animal Care and Use Committee at the University of Colorado Anschutz Medical Campus. Treatments of rats adhered to guidelines set forth in the NIH Guide for the Care and Use of Laboratory Animals. All efforts were made to minimize animal suffering and to use only the number of animals necessary to produce reliable scientific data. Experiments were performed on male and female Sprague-Dawley rats (P23-P26) obtained from Envigo (Indianapolis, IN, USA). Brain slice preparation was obtained as described previously^27^. Brain slices were immediately incubated for 30 min in the following solution (in mM): 124 NaCl, 10 D-glucose, 26 NaHCO_3_, 1.25 NaH_2_PO_4_, 4 KCl, 2 CaCl_2_, 2 MgCl_2_ at 37 °C before use in electrophysiology experiments, which were done at room temperature. During incubation, slices were constantly perfused with a gas mixture of 95 vol% O_2_ and 5 vol% CO_2_.

### Electrophysiological recordings in CeM neurons

Whole-cell recordings were performed in CeM neurons, as described previously^27^. The external solution contained (in mM): 125 NaCl, 25 D-glucose, 25 NaHCO_3_, 1.25 NaH_2_PO_4_, 2.5 KCl, 1 MgCl_2_, 2 CaCl_2_. The voltage-dependent sodium current blocker tetrodotoxin (TTX; 1 μM) was added to the extracellular medium. The internal solution for voltage-clamp experiments with Cesium (Cs) containing ATP solution consisted of the following (in mM): 110 Cs-methane sulfonate, 14 phosphocreatine, 10 HEPES, 9 EGTA, 5 Mg-ATP, and 0.3 Tris-GTP, pH adjusted to 7.15 to 7.20 with CsOH (~300 mOsm). The ATP-free internal consisted of the following (in mM): 110 Cs methane sulfonate, 10 HEPES, 9 EGTA. The AMP-PCP based internals had 3 mM AMP-PCP instead of Mg-ATP. AMP-PCP solution with BAPTA had 30 mM BAPTA as an alternative to EGTA.

### Data analysis

Current traces were analyzed using pCLAMP9 (Molecular Devices) and GraphPad Prism (GraphPad) softwares. Steady-state inactivation curves were fitted using the Boltzmann equation where I/I max = 1/(1 + exp((Vm−V_0.5_)/slope factor)). Results are presented as the mean ± SEM, and n is the number of cells. Statistical analysis were performed with two-way ANOVA combined with a Tukey post-test for multiple comparisons, excepted in Fig. 1, for which a one-way ANOVA combined with a Tukey post-test for multiple comparisons was used (*p < 0.05, **p < 0.01, ***p < 0.001).

### Chemical reagents

The CaMKII 290–309 peptide, the autocamtide-2-related inhibitory peptide (AIP) and the calcineurin inhibitory peptide (CIP) were obtained from Enzo life science (France). STO-609, BIM IX, BI 2536, BI-D1870, SU6656 and A484954 were obtained from Cayman Chemical (Interchim Inc., France). OSU-03012, AKT1/2 I, GW843682X, 5Z-oxozeaenol, CGP 57380, PF-431396, GSK 650394, CT 99021 and ML-7 were purchased from Tocris (R&D Systems Europe, France). All others compounds were obtained from Sigma (France). The control experiments were performed using the corresponding solvent.

## Supplementary information

Supplementary Information
