# Supplementary material for: A novel phospho-modulatory mechanism contributes to the calcium-dependent regulation of T-type Ca2+ channels
Source: Sci Rep. 2019 Oct 30;9:15642. doi: 10.1038/s41598-019-52194-6 (PMC6821770; doi:10.1038/s41598-019-52194-6)
Supplement: Supplementary file 1 — Supplementary Information [file 41598_2019_52194_MOESM1_ESM.pdf]

A novel phospho-modulatory mechanism contributes to the calcium-dependent regulation of T-type Ca^{2+} channels.

Jean Chemin [1,2,*], Tamara Timic Stamenic [3], Magalie Cazade [1,2], Jodie Llinares [1,2], Iulia Blesneac [1,2], Slobodan M. Todorovic [3], Philippe Lory [1,2].

[1] IGF, CNRS, INSERM, University of Montpellier, Montpellier, France; [2] LabEx 'Ion Channel Science and Therapeutics', 34094 Montpellier, France.

[3] Department of Anesthesiology, University of Colorado, Aurora, CO 80045.

Running title: Ca^{2+} / Phospho-regulation of T-channels

* Correspondence to Jean Chemin: jean.chemin@igf.cnrs.fr.

Supplementary Information

Supplementary Figure Legends

Supplementary Figure 1. Endocytosis and lysosomal degradation are not involved in the activity-dependent $Ca_v3.3$ current inhibition.

(A-D) Examples of $Ca_v3.3$ currents elicited by the 1 Hz stimulation protocol during 40 s for the 1st and the 3rd stimulation for cells expressing Dynamin WT (A), Dynamin K44A (B), AP-2 (C), and for cells treated with 100 nM bafilomycin for 4-5 hours (D). **(E)** Amplitude of the $Ca_v3.3$ current at the beginning (1 s) and after 40 s obtained at the 1st and the 3rd stimulation

normalized to the initial current amplitude I (1s 1st). Asterisks indicate significant difference in the current amplitude, as compared to the initial current. In addition, the current inhibition (red bars) and recovery (blue bars) as function of the treatment was statistically compared to the respective control condition, as indicated. The number of cells tested is indicated into brackets. (*n.s.* : non-significant).

Supplementary Figure 2. Alkaline phosphatase induces the inhibition of the $Ca_v3.3$ current and abolishes the activity-dependent $Ca_v3.3$ current inhibition.

(A) Effect of the 1 Hz frequency stimulation on the Cav3.3 current before (1st) and after (3rd) ~20 mn dialysis of an intracellular solution containing 100 U/ml alkaline phosphatase (AP). **(B)** Amplitude of the $Ca_v3.3$ current at the beginning (1 s) and after 40 s obtained at the 1st and the 3rd stimulation normalized to the initial current amplitude I (1s 1st). **(C)** Inactivation kinetics of the $Ca_v3.3$ current at the beginning of the stimulation (1 s) and after 40 s stimulation obtained for the 1st and the 3rd stimulation. **(D)** Steady-state inactivation curves of the $Ca_v3.3$ current obtained from control cells or cells dialyzed with AP. Asterisks indicate significant difference in the current amplitude (B) and inactivation kinetics (C), as compared to the initial current. In addition, variation in the current amplitude (B) and in inactivation kinetics (C) as function of the AP treatment was statistically compared to the respective control condition, as indicated. The number of cells tested is indicated into brackets. (*n.s.* : non-significant).

Supplementary Figure 3. AMP-PCP does not induce rundown of the $Ca_v3.3$ current at a low frequency of stimulation.

(A) Time-course of the $Ca_v3.3$ current in cells dialyzed with 3 mM ATP or 3 mM AMP-PCP during low frequency of stimulation (0.0055 Hz, left panel) followed by the fast stimulation protocol (1Hz), which was performed 3 times (right panel). (*n.s.* : non-significant).

Supplementary Figure 4. The modulation of the $Ca_v3.3$ current at fast frequency stimulation is insensitive to the reducing agent dithiothreitol.

(A-B) Typical $Ca_v3.3$ currents elicited by the 1 Hz stimulation protocol during 40 s for the 1st and the 3rd stimulation in the control cells (A) and in the cells dialyzed with 1 mM dithiothreitol (B, DTT). **(C)** Amplitude of the $Ca_v3.3$ current at the beginning (1 s) and after 40 s obtained at the 1st and the 3rd stimulation normalized to the initial current amplitude I (1s 1st). **(D)** Inactivation kinetics of the $Ca_v3.3$ current at the beginning of the stimulation (1 s) and after 40 s stimulation obtained for the 1st and the 3rd stimulation. Asterisks indicate significant difference in the current amplitude (C) and inactivation kinetics (D), as compared to the initial current. In addition, variation in the current amplitude (C) and in inactivation kinetics (D), as function of the DTT treatment, was statistically compared to the respective control condition, as indicated. The number of cells tested is indicated into brackets. (*n.s.* : non-significant).

Supplementary Figure 5. The activity-dependent $Ca_v3.3$ current modulation does not involve PKA, PKC, Rho-associated kinase, phospholipase C, Gβγ and PI3K/PI4K.

(A) Effect of db-cAMP (1 mM, 15 minutes incubation), PMA (1 µM, 15 minutes incubation), chelerythrine (1 µM, 4 hours incubation), BIM IX (5 µM in the patch pipette), fasudil (10 µM, 2 hours incubation), U73122 (10 µM in the patch pipette), edelfosine (10 µM in the patch pipette), PTX (0.5 µg/ml, overnight incubation), βARK expression, wortmannin (10 µM, 2 hours incubation) and CPA (20 µM, 30 minutes incubation) on the inhibition of $Ca_v3.3$ current induced by the 1 Hz stimulation protocol obtained at the 3rd stimulation. The effects of the drugs were compared to the percent of inhibition of the $Ca_v3.3$ current obtained in the matched control condition. **(B)** Effect of db-cAMP, PMA, chelerythrine, BIM IX, fasudil, U73122, edelfosine, PTX, βARK expression, wortmannin and CPA on the recovery of $Ca_v3.3$ current after the 1 Hz

stimulation protocol obtained at the beginning of the 3rd stimulation. The effects of the drugs were compared to the percent of recovery of the $Ca_v3.3$ current obtained in the matched control condition. The number of cells tested is indicated into brackets. (*n.s.* : non-significant).

Supplementary Figure 6. Effect of various kinase inhibitors on the activity-dependent $Ca_v3.3$ current modulation

(A) Effect of OSU-03012 (10 µM in the patch pipette), AKT 1/2 inhibitor (1 µM, 1 hour incubation), GW843682X (10 µM, 1 hour incubation), BI 2536 (1 µM, 1 hour incubation), BI-D1870 (5 µM, 1 hour incubation), 5Z-oxozeanol (1 µM, 1 hour incubation), CGP 57380 (10 µM, 1 hour incubation), PF-431396 (10 µM in the patch pipette), GSK 650394 (10 µM, 1 hour incubation), CT 99021 (1 µM, 1 hour incubation), SU6656 (1 µM, 1 hour incubation), A484954 (10 µM, 1 hour incubation), and ML-7 (20 µM in the patch pipette) on the inhibition of $Ca_v3.3$ current induced by the 1 Hz stimulation protocol obtained at the 3rd stimulation. The effects of the drugs were compared to the percent of inhibition of the $Ca_v3.3$ current obtained in the matched control condition. **(B)** Effect of the kinase inhibitors on the recovery of $Ca_v3.3$ current after the 1 Hz stimulation protocol obtained at the beginning of the 3rd stimulation. The effects of the drugs were compared to the percent of recovery of the $Ca_v3.3$ current obtained in the matched control condition. The number of cells tested is indicated into brackets. (*n.s.* : non-significant).

Supplementary Figure 7. A schematic illustration of the hypothesized mechanisms involved in the T-type current regulation at fast frequency stimulation.

Stimulation of the $Ca_v3.3$ and $Ca_v3.1$ (but not $Ca_v3.2$) channels at fast frequency (1 Hz) induces a rise in local submembrane Ca^{2+}. This rise in submembrane Ca^{2+} induces the activation of a putative phosphatase, whose activity produces the inhibition of the T-type current. Conversely,

the recovery of the current depends on the activity of a yet unidentified kinase. Whether the Ca_v3 channel is directly phosphorylated or modulated via an intermediate protein remains unclear and requires further investigation. Action of various inhibitors used in this study is indicated.

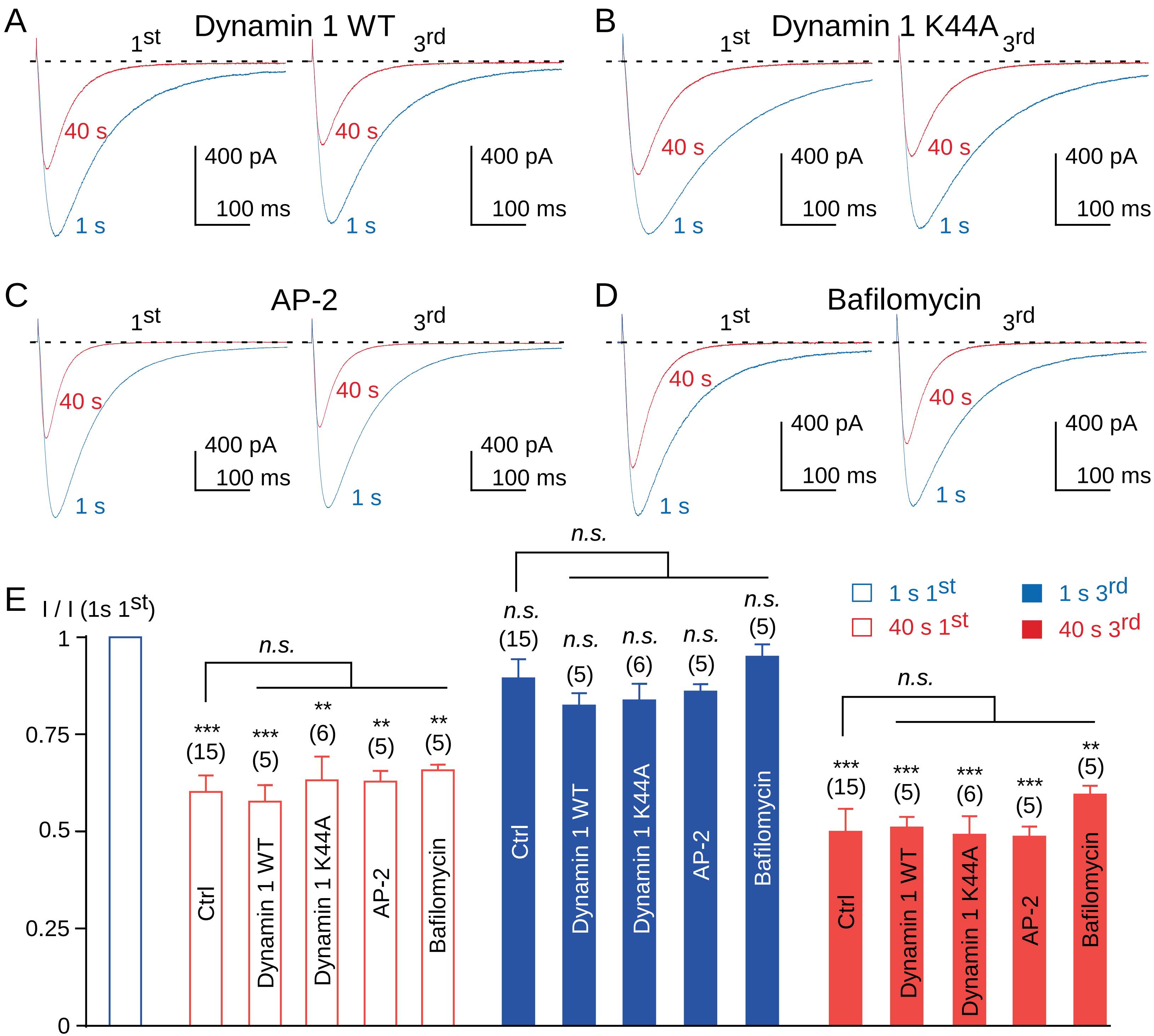

Supplementary Figure 1

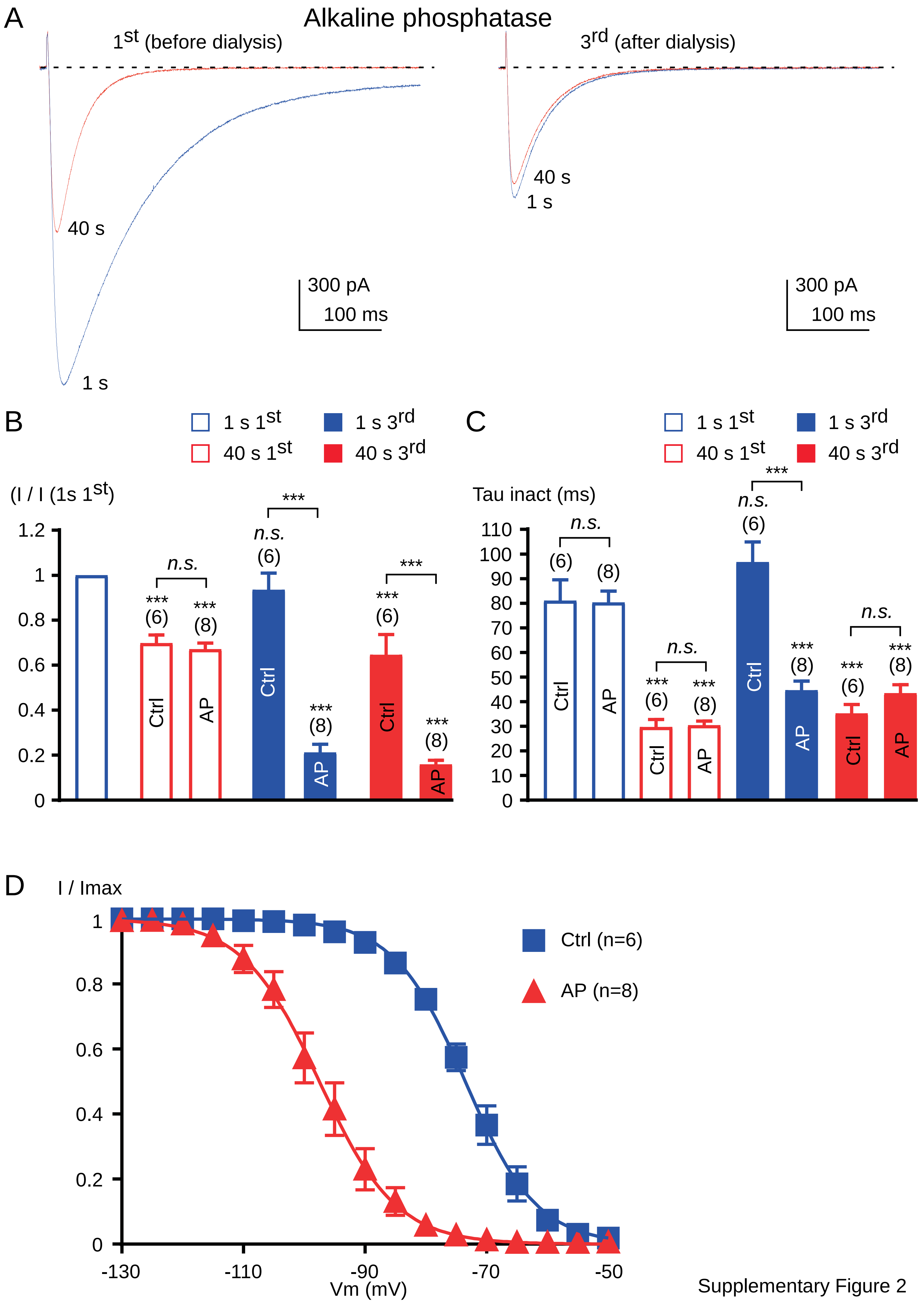

Supplementary Figure 2

A

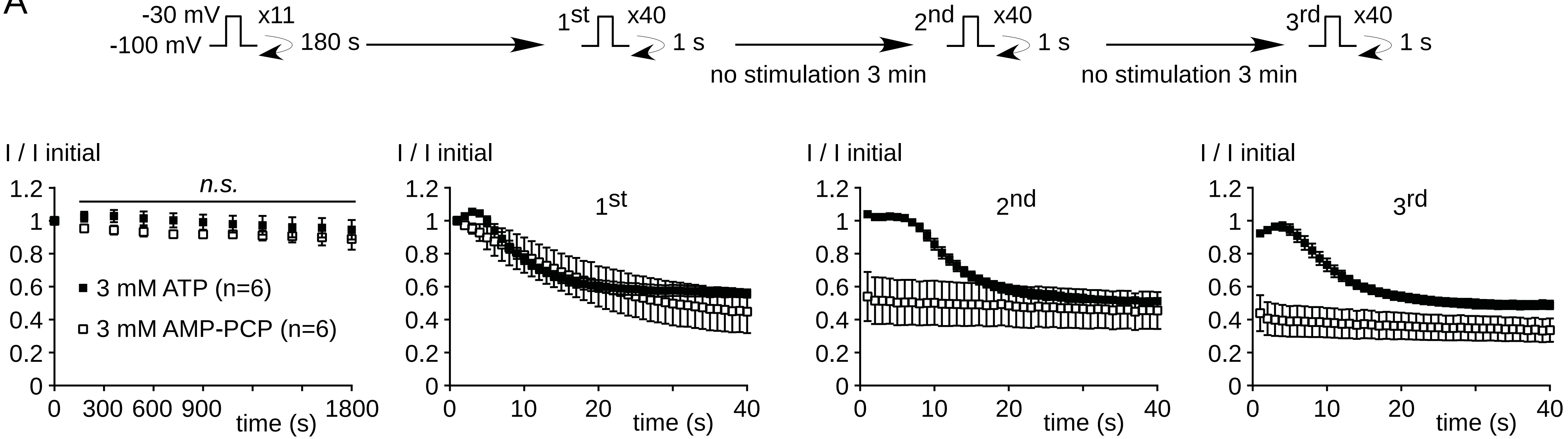

Supplementary Figure 3

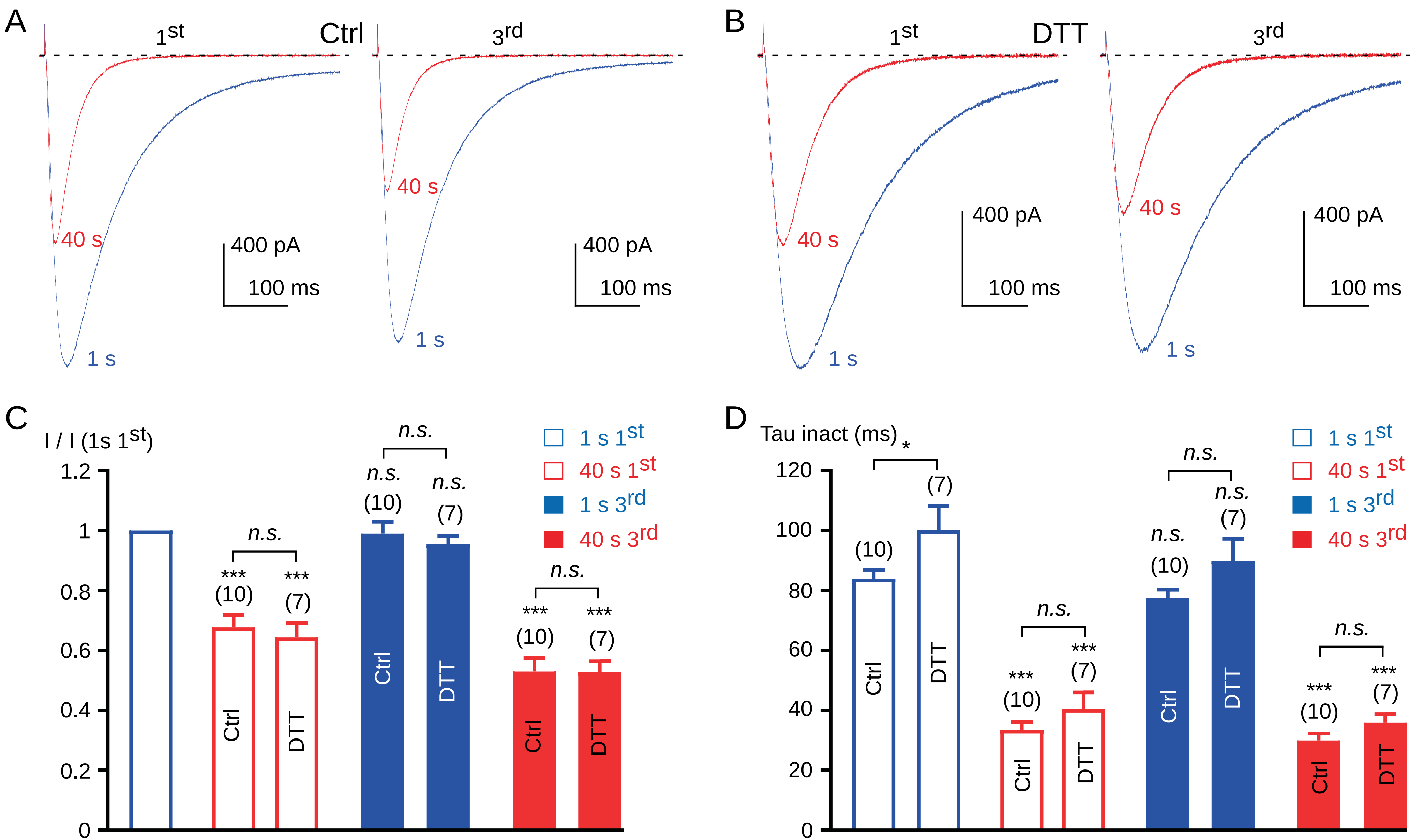

Supplementary Figure 4

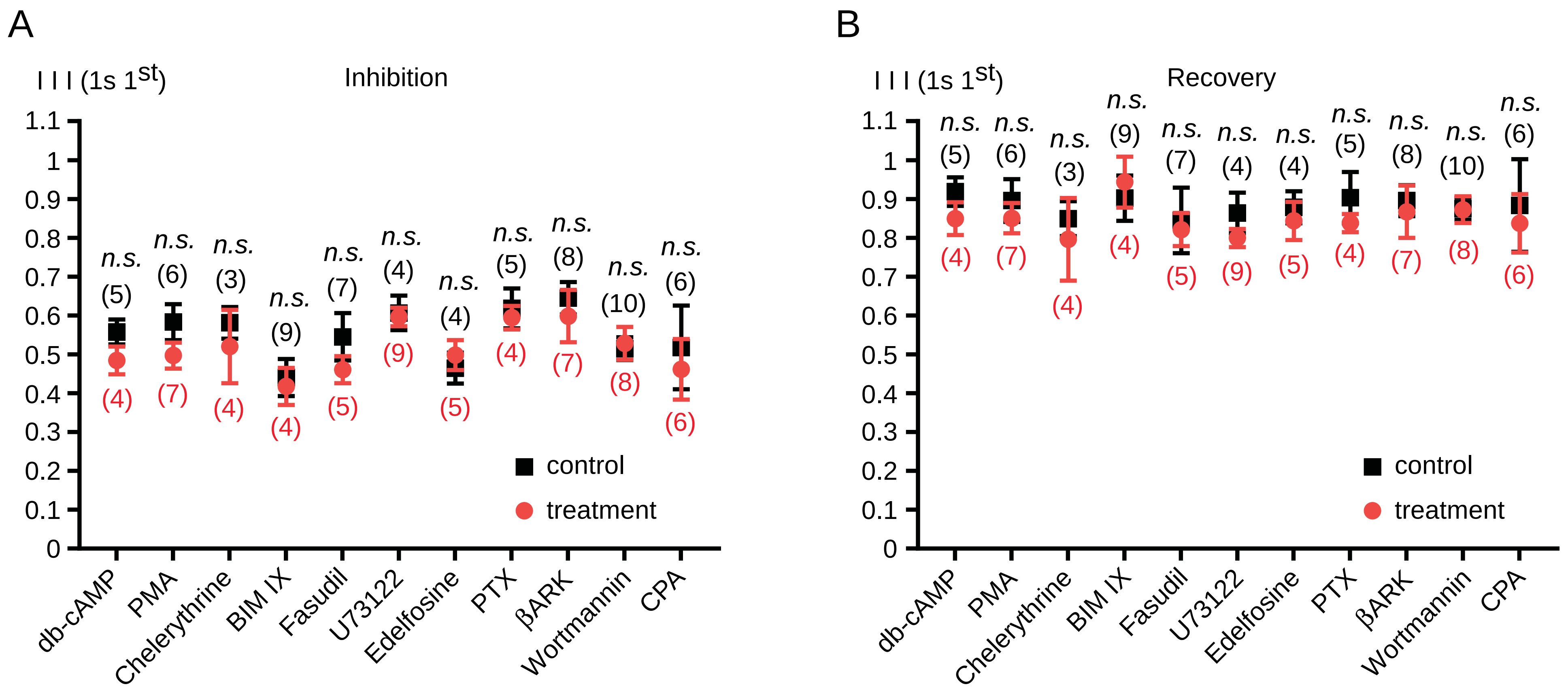

Supplementary Figure 5

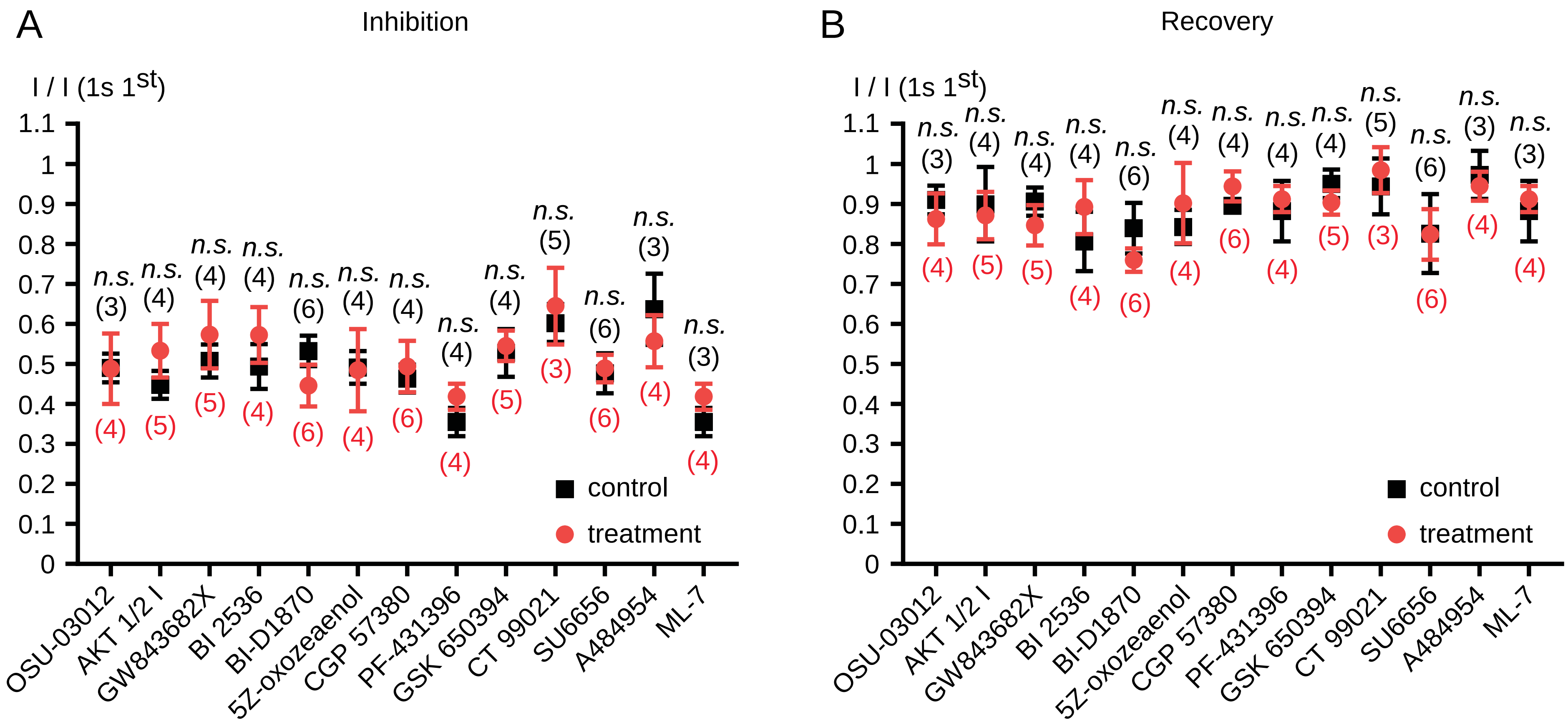

Supplementary Figure 6

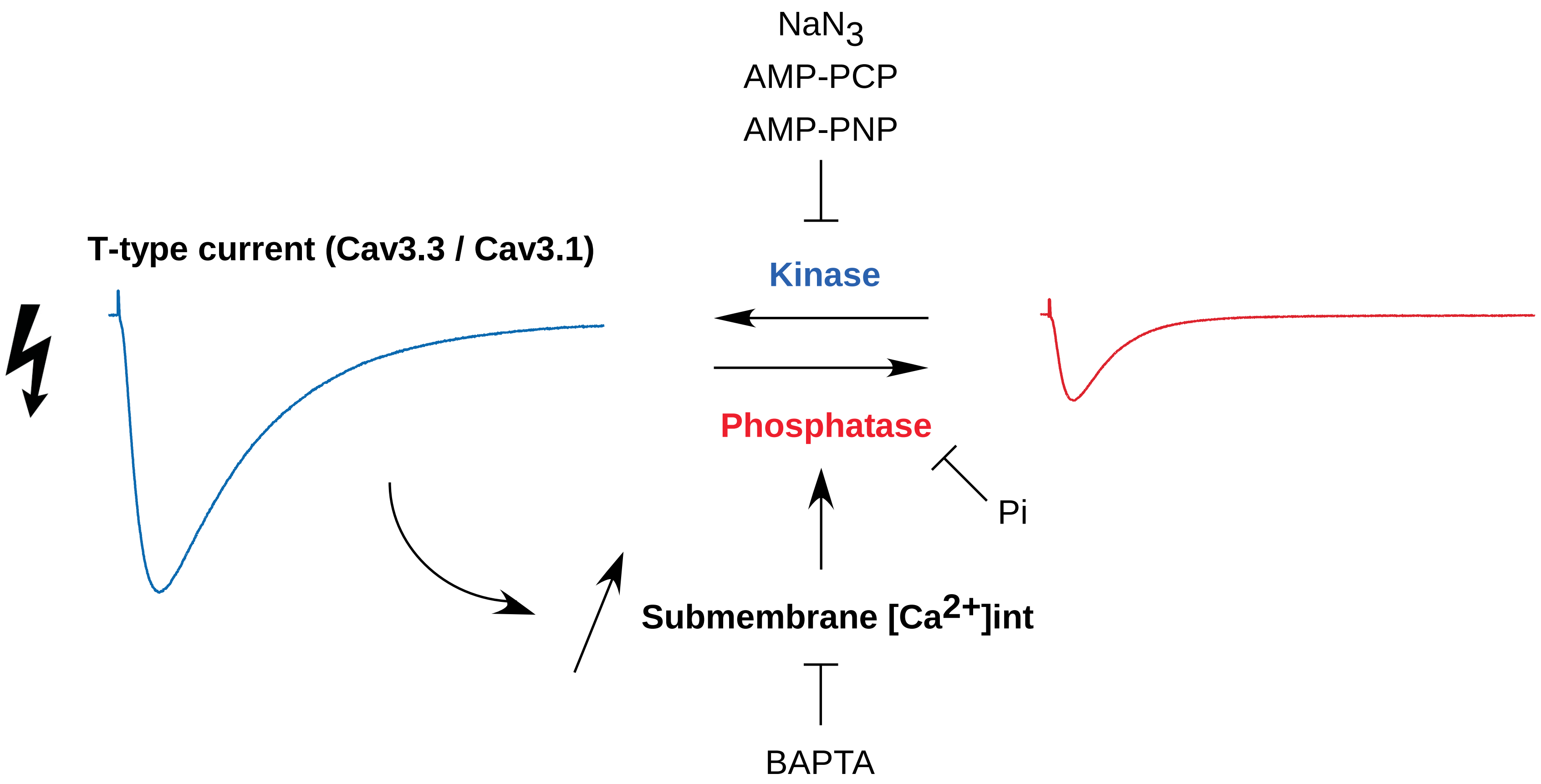

Supplementary Figure 7
